# Tunable Optical Properties of Amorphous-Like Ga_2_O_3_ Thin Films Deposited by Electron-Beam Evaporation with Varying Oxygen Partial Pressures

**DOI:** 10.3390/nano10091760

**Published:** 2020-09-06

**Authors:** Shijie Li, Chen Yang, Jin Zhang, Linpeng Dong, Changlong Cai, Haifeng Liang, Weiguo Liu

**Affiliations:** Shaanxi Province Key Lab of Thin Films Technology and Optical Test, School of Photo-Electrical Engineering, Xi’an Technological University, Xi’an 710021, China; lishijie@xatu.edu.cn (S.L.); zhangjin@xatu.edu.cn (J.Z.); lpdong@xatu.edu.cn (L.D.); caichanglong@xatu.edu.cn (C.C.); lianghaifeng@xatu.edu.cn (H.L.); liuweiguo@xatu.edu.cn (W.L.)

**Keywords:** Ga_2_O_3_ film, electron-beam evaporation, optical properties

## Abstract

Ga_2_O_3_ thin films were fabricated by the electron-beam evaporation technique at a varying oxygen partial pressure from 0 to 2.0 × 10^−2^ Pa. The effect of oxygen partial pressure on the crystalline structure and optical properties of the Ga_2_O_3_ films was analyzed using sophisticated techniques including X-ray photoelectron spectroscopy (XPS), X-ray diffraction (XRD), Raman spectroscopy, spectroscopic ellipsometry, ultraviolet-visible spectroscopy and a laser-induced damage test system. The correlation between the oxygen partial pressure and the film’s properties in optics and materials were investigated. XRD and Raman revealed that all films were amorphous in spite of applying a varying oxygen partial pressure. With the change of oxygen partial pressure, XPS data indicated that the content of oxygen in the Ga_2_O_3_ films could be broadly modulable. As a result, a changeable refractive index of the Ga_2_O_3_ film is realizable and a variable blue-shift of absorption edges in transmittance spectra of the films is achievable. Moreover, the damage threshold value varied from 0.41 to 7.51 J/cm^2^ according to the rise of oxygen partial pressure. These results demonstrated that the optical properties of Ga_2_O_3_ film can be broadly tunable by controlling the oxygen content in the film.

## 1. Introduction

Gallium oxide (Ga_2_O_3_) possesses diverse crystalline phases such as α-, β-, γ-, δ-, ε-Ga_2_O_3_ and so on [1]. Due to the high direct gap of 4.9 eV, Ga_2_O_3_ is reckoned to be a sort of wide band gap semiconductor material [2]. Besides that, the high chemical and thermal stability of Ga_2_O_3_ make itself a favorite film for applying in a harsh environment [3].

Broad investigations on Ga_2_O_3_ material with multiple forms such as single crystals bulk, nanostructures and thin films have been conducted in recent years [4,5,6]. Most of them focus on the thin-film structures due to their low cost and applicability. The preparation of Ga_2_O_3_ thin films have been finalized by low-pressure chemical vapor deposition (LPCVD) [7], mist chemical vapor deposition (mist-CVD) [8], metal-organic chemical vapor deposition (MOCVD) [9], halide vapor phase epitaxy (HVPE) [10], pulsed laser deposition (PLD) [11], hydrothermal [12], sol-gel [13] and sputtering [14]. Ga_2_O_3_ films are employed for fabricating various component, such as solar-blind ultraviolet photodetectors, phosphor, transparent conductors, transparent electronic devices and gas sensors [15,16,17]. However, the related investigations are still limited as far as the potential optical-coating application for the Ga_2_O_3_ thin film is concerned.

Thus, our investigation intentionally planned to focus on the optical properties of the amorphous-like Ga_2_O_3_ thin films commonly applied in optics field, which were deposited by a low-cost and larger-scale deposition method of electron-beam evaporation. Considering the optical properties of Ga_2_O_3_ thin films are greatly affected by oxygen partial pressure, the properties of the thin film, such as stoichiometry, microstructure, and optical characteristics, have been comprehensively analyzed under the different oxygen partial pressures. This investigation would be beneficial for exploring our new knowledge of fundamental properties of Ga_2_O_3_ films.

## 2. Experimental Details

Ga_2_O_3_ granules, approximately 3 mm in diameter, was used as staring materials with a high purity of 99.995%. Through the use of an electron-beam evaporation deposition system (ZZS500-1/G, Rankuum Machinery Ltd., Chengdu, China), the staring materials were evaporated to form Ga_2_O_3_ films on Si and the fused quartz substrates, respectively. Before the cleaned substrates were clamped on the rotating workpiece-disk in the evaporation chamber, all substrates were cleaned by a mixed solution of alcohol and ether, and then rinsed in deionized water. As the base vacuum pressure reached 3 × 10^−3^ Pa, the starting materials were heated by electron beam gun and Ga_2_O_3_ film were deposited on the substrates at 100 °C. At the same time, high purity (99.999%) oxygen gas was passed though the vacuum chamber with different fluxes to form oxygen partial pressure varying from 0 to 2.0 × 10^−2^ Pa with an interval of 4.0 × 10^−3^ Pa. Moreover, using an optical monitor, the deposition rate was kept at 0.3–0.4 nm/s during the whole evaporation process.

The as-deposited Ga_2_O_3_ films were analyzed by X-ray photoelectron spectroscopy (XPS, K-Alpha, Thermo Fisher, Waltham, MA, USA) with monochromatized Al-Kα radiation (*hν* = 1486.6 eV) as an excitation source to obtain the elemental composition and chemical states of elements. The data of phase structure and crystallinity from the films were gathered by X-ray diffractometer (XRD) with a small-angle X-ray scattering method. Raman spectra were collected by a Horiba XploRA PLUS (Kyoto, Japan) surveying near-field optical microscope under an 532 nm laser excitation.

**Figure 1:**
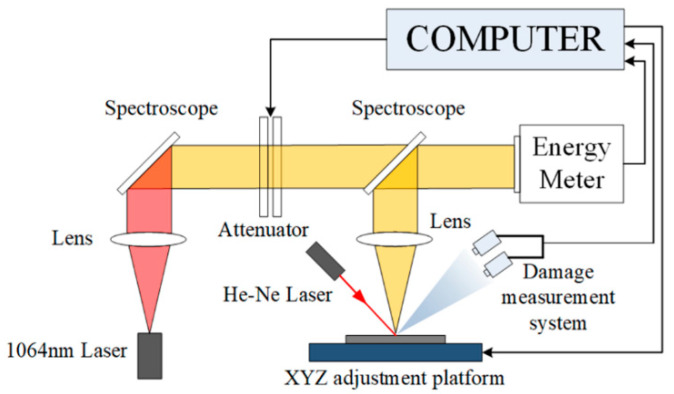
The schematic diagram of the laser-induced testing system.

The refractive indexes and thicknesses of the films were measured by the M-2000UI spectroscopic ellipsometry (J.A. Woollam, Lincoln, NE, USA). The transmission spectra were recorded by the UV3150 spectrophotometer (Shimadzu, Kyoto, Japan) over the wavelength range from 200 to 1200 nm. Laser-induced damage testing was conducted in the “1-on-1” regime stemming by the international standard ISO 11254. Figure 1 shows a schematic diagram of the laser-induced testing system. A Q-switched Nd: YAG laser of a 1064-nm wavelength and a 10 nm pulse width operating was operated in a single mode of TEM_00_. Through laser focusing, a far-field circular Gaussian beam of 0.8 mm in diameter was obtained, which had a nearly flat-top intensity distribution. The thin film to be measured was clamped into the sample holder on a second-dimensional stage which could be precisely driven along X and Y orientations by a stepping motor. The incident angle was slightly deviated from the normal incidence by 3° to avoid interference from reflection of the sample surface. The attenuator was applied to adjust the laser energy varying from 1 to 50 J/cm^2^, which was exerted on the sample for damage measurement. At the same time, an energy meter was adopted to gauge the pulse energy from a split portion of the incident beam. Ten evenly increasing laser energy levels were loaded on the film sample by the international standard. At each level, the pulses were exerted 10 times at different positions on the sample surface. By applying the method of phase contrast microscopy, damage onsets were examined and distinguished by a microscope loaded with a charge couple device camera. Correspondingly, the damage possibility of each level of laser energy can be evaluated by 10 measurements. It should also be noted that the laser-induced damage threshold here was defined as the intersection point between laser energy axis and the fitting line of damage probabilities obtained at various energy levels. During the whole period of measurement, a charge couple device camera was employed to detect the onsets of damage.

## 3. Results and Discussions

### 3.1. Chemical Structures of Ga_2_O_3_ Films

**Figure 2:**
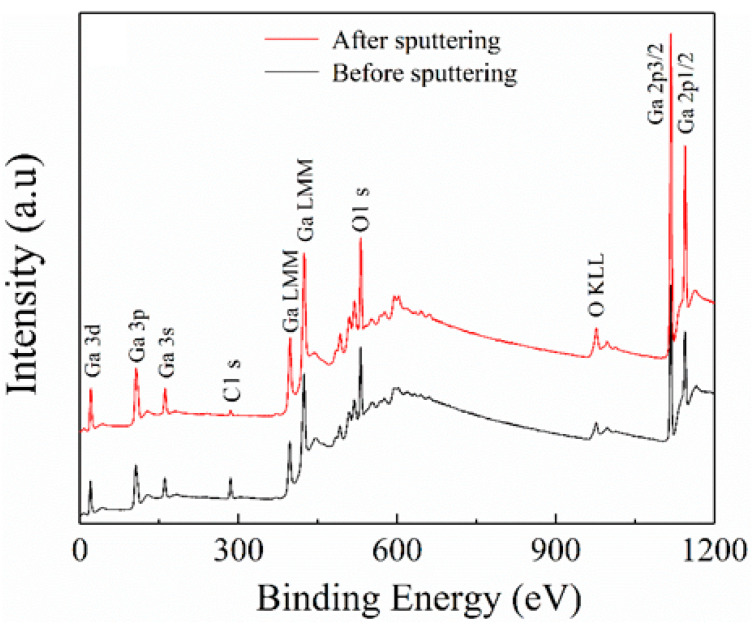
The X-ray photoelectron spectroscopy (XPS) survey spectra of the Ga_2_O_3_ film deposited at oxygen partial pressure of 0 Pa before and after the etching process.

All the Ga_2_O_3_ films deposited at various oxygen partial pressure were subject to the XPS measurement for analyzing the chemical valences of films. To avoid the influence from the adventitious hydrocarbon contamination caused by the exposure of samples to ambient air, all the films were sputtered by 3 keV Ar^+^ for 30 s before the XPS measurement. The XPS survey spectra of the Ga_2_O_3_ film deposited at oxygen partial pressure of 0 Pa, before and after the etching process, are displayed in Figure 2. The C 1s standard peak is located at 284.6 eV. An obvious Ga 3d signal peak is located at the binding energy of 20 eV, which is in line with the reported result of Ga_2_O_3_ [18,19]. The peaks located at 106 and 161 are, respectively, related to Ga 2p and Ga 2s. The peak of 531 eV is connected to the core level of O 1s. Moreover, the peaks of 398 and 425 eV correspond to Ga LMM Auger line, and the peak of 977eV are associated with O KLL Auger line. Finally, the last two peaks located at the binding energy of 1118 and 1144 eV match with the core levels of Ga 2p3/2 and Ga 2p1/2, respectively [18,20]. Moreover, no other obvious signal peak is found. After sputtering, the intensities of all signal peaks are dramatically increased except the C 1s, whereas C 1s is immensely decreased in peak intensity. It reveals that the surface contaminations have been effectively cleared out by the sputtering of 30 nm. The results discussed above are also present in other films’ survey spectra.

**Figure 3:**
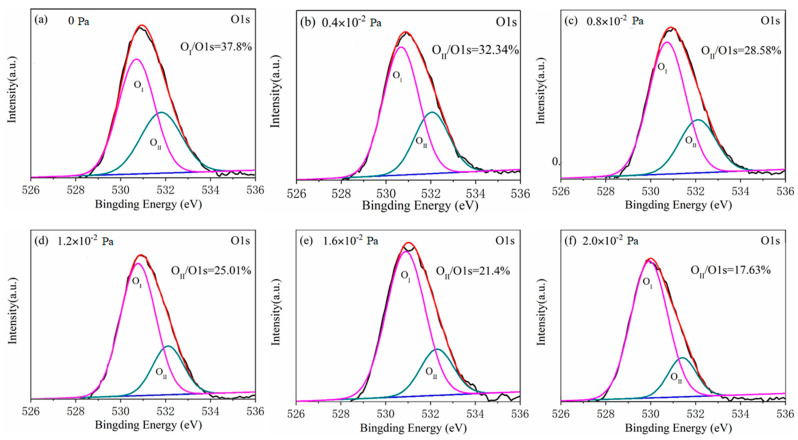
The asymmetrical high-resolution XPS spectra of O 1s corresponding to films of various oxygen partial pressure of (**a**) 0 Pa, (**b**) 0.4 × 10^−2^ Pa, (**c**) 0.8 × 10^−2^ Pa, (**d**) 1.2 × 10^−2^ Pa, (**e**) 1.6 × 10^−2^ Pa and (**f**) 2.0 × 10^−2^ Pa.

**Figure 4:**
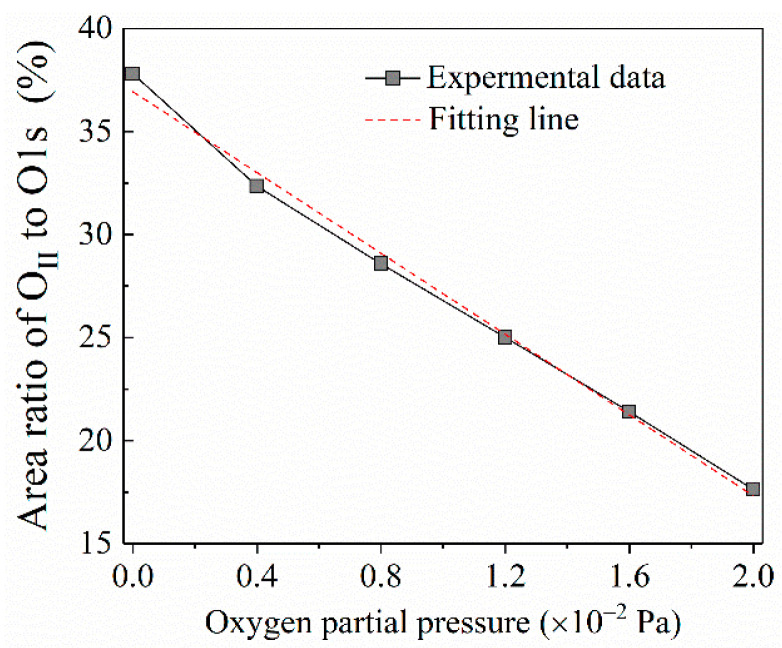
The relation between the area ratio of O_II_ peak to O 1s peak and oxygen partial pressure.

It is noteworthy that the high resolution XPS spectra of O 1s peaks from all tested films differ considerably. The asymmetrical high-resolution XPS spectra of O 1s corresponding to the films of all given oxygen partial pressure are shown in Figure 3. Each high-resolution spectrum is deconvolved into two peaks respectively located at approximately 530 eV (O_I_ peak) and 532 eV (O_II_ peak) by the Gaussian fitting method. Correspondingly, the lower binding energy peak should be related to the oxide (Ga–O–Ga) bonds, whereas the higher binding peak is associated with oxygen vacancy in Ga_2_O_3_ film [21]. The area ratio changes of O_II_ peak to O 1s peak from 37.80% to 17.63% with the oxygen partial pressure increasing from 0 to 2.0 × 10^−2^ Pa with an interval of 4 × 10^−3^ as displayed in Figure 4. This shows that the oxygen vacancy in Ga_2_O_3_ film during depositing process can be effectively controlled by the increase of oxygen partial pressure. From the fitting result, it can be observed that there is a linear relationship between the area ratio and the oxygen partial pressure, which can be described by the formula as following:

(1) y=−0.098x+0.369

where *y* represents the area ratio of O_II_ peak to O 1s peak; *x* stands for the oxygen partial pressure. Applying the formula, it can be deduced that a zero-ratio corresponding to a non-vacancy situation would be approached when the oxygen partial pressure continuously increases to 3.76 × 10^−2^ Pa. However, during the deposition process of the EBV (electron-beam evaporation) method, the vacuum degree less than 5 × 10^−2^ Pa is necessary for the normal functioning of an electron beam gun. In addition, a pressure surge from the thermal effect produced by running the gun should be considered. Therefore, the upper limit of 3.0 × 10^−2^ Pa has to be maintained in practice, which hinders further increase of oxygen partial pressure.

### 3.2. Crystalline Structures of Ga_2_O_3_ films

**Figure 5:**
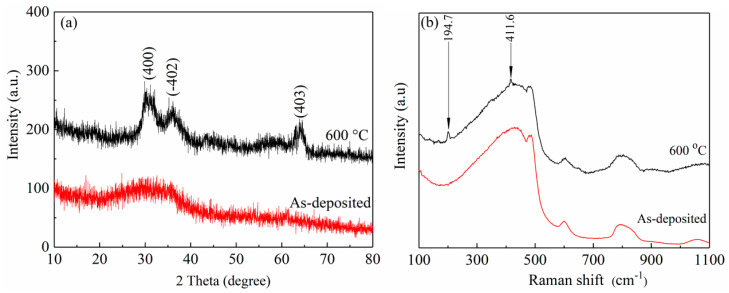
(**a**) X-ray diffraction (XRD) spectra and (**b**) Raman spectra of the as-deposited film and its 600 °C annealed film deposited at 1.2 × 10^−2^ Pa.

Crystalline structure analysis shows all the as-deposited films evaporated at different oxygen partial pressure have an amorphous structure. The XRD spectra of the as-deposited film and its 600 °C-annealed film, both corresponding to the partial pressure of 1.2 × 10^−2^ Pa, are presented in Figure 5a. The non-crystalline characteristic of the as-deposited film is exposed by the detected envelope-peak from 20° to 40° on the XRD spectrum. After the same film is exerted by an annealing process at 600 °C for 1 h, three weak diffraction peaks are detected at 30.3°, 36.3°, and 64.5° on the XRD spectrum, corresponding well to (400), (−402) and (403) planes of monoclinic β-Ga_2_O_3_ (JCPDS#41-1103), indicating the onset of film crystallization.

For a further crystalline structure study, Raman spectroscopy is carried out on the as-deposited and the annealed films at 600 °C for 1 h. Raman spectra collected from the as-deposited Ga_2_O_3_ film and the 600 °C-annealed film at the room-temperature are displayed in Figure 5b, respectively. In the range from 100 to 1000 cm^−1^, there are no more obvious peaks in the spectrum of the as-deposited film except four broad characteristic peaks of the fused quartz substrates. However, the spectrum of the 600 °C-annealed film possesses two extra peaks of active modes around 194.7 (A_g_) and 411.6 (A_g_) cm^−1^. These two Raman peaks with weak intensity in the spectrum of 600 °C-annealed film might be attributed to the poor crystalline quality of the film, which is in accordance with the results of XRD. Moreover, the lower frequency modes of 194.7 cm^−1^ are connected with vibration and translation of tetrahedra-octahedra chains, the higher frequency modes of 411.6 cm^−1^ are connected with deformation of Ga_2_O_6_ octahedra.

### 3.3. Optical Properties of Ga_2_O_3_ Films

**Figure 6:**
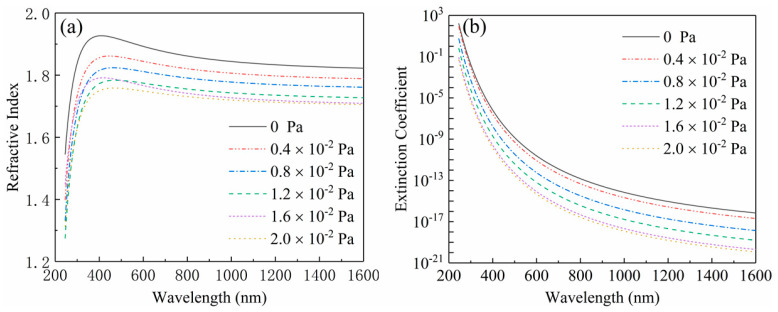
The dispersion curves of (**a**) the refractive indexes and (**b**) extinction coefficients from the films deposited at different oxygen partial pressure.

**Figure 7:**
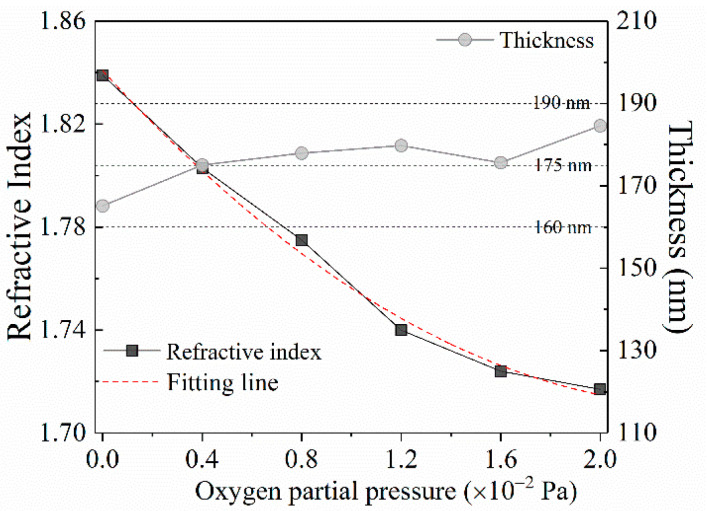
The change trend of refractive index and thickness of the films with increase of oxygen partial pressure.

As shown in Figure 6, the refractive indexes and the extinction coefficients of the films deposited at different oxygen partial pressures were measured by ellipsometry in the band range of 250–1600 nm. The Figure 6a displays the dispersion curves of the refractive indexes. It is shown that all dispersion curves ascend along with the increase of wavelength in the near-ultraviolet range (240 nm < *λ* < 400 nm), and then descend in the visible and near-infrared range (400 nm < *λ*< 1650 nm), which implies that the films have a stronger absorption characteristic in the ultraviolet range and have a better transparent property in the visible and near-infrared range. This result can be further proved by the change trend occurrence in the dispersion curves of extinction coefficients plotted in the Figure 6b. Moreover, each dispersion curve declines lower than another with the increase of oxygen partial pressure, which demonstrates that the refractive index of the Ga_2_O_3_ films can be modulated through varying oxygen partial pressure. As shown in the Figure 6, the refractive indexes were 1.839, 1.803, 1.775, 1.740, 1.724 and 1.717 at the wavelength of 1046 nm corresponding to the oxygen partial pressure of 0, 0.4 × 10^−2^, 0.8 × 10^−2^, 1.2 × 10^−^^2^, 1.6 × 10^−2^ and 2 × 10^−2^ Pa, respectively. With the increase of oxygen partial pressure, the change trend of the refractive index could be properly described by a fitting line shown in the Figure 7, which is expressed as the following quadratic formula:

(2) $$y=0.02x^{2}-0.11x+1.84$$

where *y* represents the refractive index; and *x* stands for the oxygen partial pressure. Hence, the refractive index relating to arbitrary pressure can be obtained by the formula. The curve of thicknesses from Ga_2_O_3_ films deposited at different oxygen partial pressure is plotted in the Figure 7. It indicates that the consistency in thickness is controlled well for all films with a deviation less than ±15 nm.

**Figure 8:**
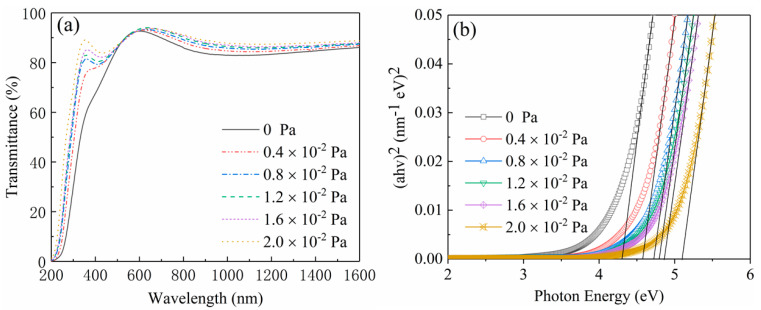
The optical transmission spectra (**a**) and the relation plots between (*αhv*)^2^ and photon energy *hν* (**b**) of the Ga_2_O_3_ thin films deposited at different oxygen partial pressures.

The optical transmission spectra of the Ga_2_O_3_ films measured from 200 to 1600 nm at room temperature are revealed in Figure 8a. All films are transparent in the visual and near-infrared range. The similar interference ripples in these spectra indicate that the thicknesses of all films are very close, which is highly consistent with the result obtained in Figure 7. As shown in Figure 8a, the optical transmission of Ga_2_O_3_ films in the visible and the near-ultraviolet (UV) regions is apparently improved by increasing the oxygen partial pressure. With the increase of the oxygen partial pressure, the edge of absorption shows a characteristic of dramatically blue-shifted at the same time. Figure 8b presents the relation plots between (*αhv*)^2^ and photon energy *hν* of the Ga_2_O_3_ thin films deposited at different oxygen partial pressure. The optical band-gap values of different films are derived from extrapolating the linear portion of (*αhv*)^2^ to *hν* axis [21]. It can be seen that the optical band-gap value of Ga_2_O_3_ film varies from 4.3 to 5.1 eV corresponding to the increase of oxygen partial pressure from 0 to 2.0 × 10^−2^ Pa. This means that a modulable optical band-gap can be obtained by varying the oxygen partial pressure during the deposition process.

**Figure 9:**
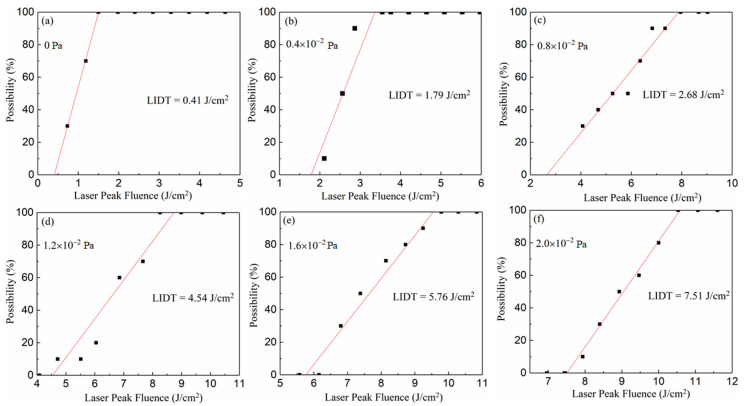
The relationship between laser energy and damage probabilities of different films deposited at (**a**) 0 Pa, (**b**) 0.4 × 10^−2^ Pa, (**c**) 0.8 × 10^−2^ Pa, (**d**) 1.2 × 10^−2^ Pa, (**e**) 1.6 × 10^−2^ Pa and (**f**) 2.0 × 10^−2^ Pa.

**Figure 10:**
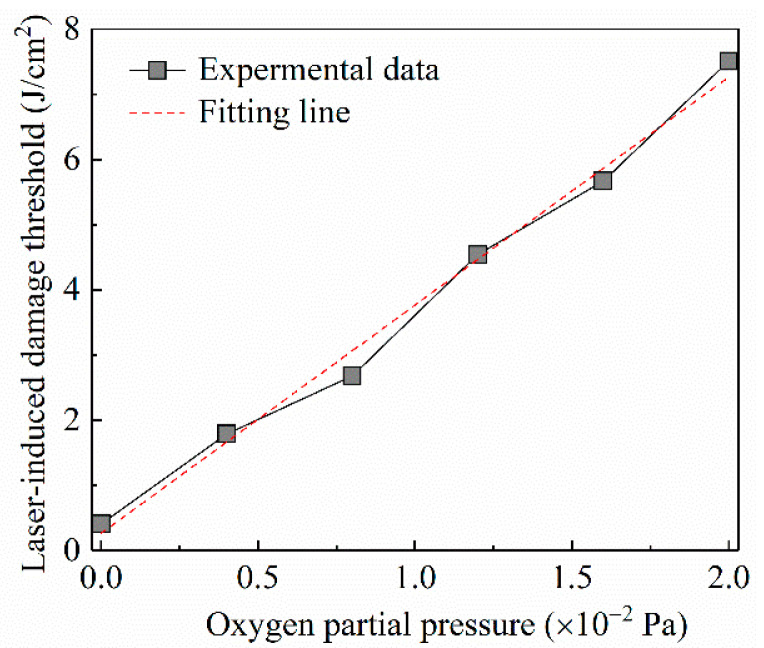
The changing trend of laser-induced damage thresholds of the Ga_2_O_3_ films deposited at different oxygen partial pressure.

The laser-damage characteristic of films fabricated at different oxygen partial pressure can be evaluated by the laser-induced damage threshold, which is measured on the basis of the international standard of ISO11254. Ten levels of laser energy with the same interval were shot on the film. For each energy level, the tests were undertaken 10 times on different positions of the films’ surfaces. Correspondingly, the damage probabilities of each energy level were obtained from the 10 tests. Finally, the laser-induced damage thresholds of different films were derived by fitting the relationship between laser energy and damage probabilities as separately shown in Figure 9a–f. This demonstrates that the LIDT (laser-induced damage threshold) of the Ga_2_O_3_ film increases from 0.41 to 7.51 J/cm^2^ with the rise of oxygen partial pressure from 0 to 2.0 × 10^−2^ Pa. With the rise of oxygen partial pressure, the changing trend of laser-induced damage thresholds of the Ga_2_O_3_ films is displayed in Figure 10, which closely conforms to a linear formula expressed as follows:

(3) y=3.5x+0.27

where *y* represents the refractive index; and *x* stands for the oxygen partial pressure. This implies that the laser-induced damage threshold of Ga_2_O_3_ film would be dramatically improved due to less oxygen vacancy occurring in the deposition process of higher oxygen partial pressure.

## 4. Conclusions

Under different oxygen partial pressure, the amorphous-like Ga_2_O_3_ thin films can be deposited on Si and quartz substrates by electron beam evaporation. It can be found that the loss of oxygen in Ga_2_O_3_ film can be broadly controlled by varying the oxygen partial pressure. As a result, with the varying of depositing temperature, optical properties such as the refractive index, transmittance and optical band-gap can be modulated in a major range to meet different requirements. Also, the laser-induced damage property of the thin film possessed good adjustability. These results enrich our understanding of many basic properties of Ga_2_O_3_ films, and provide more practical guidance for the fabrication and application of Ga_2_O_3_ films in optical areas.
